# “Inside-out” technique to allow conduction system pacing in superior vena cava obstruction

**DOI:** 10.1016/j.hrcr.2024.11.002

**Published:** 2024-11-12

**Authors:** Alphonsus C. Liew, Nadeev Wijesuriya, Felicity de Vere, Sandra Howell, Stephen Black, Christopher Aldo Rinaldi

**Affiliations:** ∗ School of Biomedical Engineering and Imaging Sciences, King’s College London, London, United Kingdom, and Department of Cardiology, Guy’s and St Thomas’ NHS Foundation Trust, London, United Kingdom; † Department of Vascular Surgery, Guy’s and St Thomas’ NHS Foundation Trust, London, United Kingdom

**Keywords:** Inside-out, Central venous occlusion, Conduction system pacing, Cardiac resynchronization therapy, Implantable cardiac defibrillator

**Key Teaching Points**

• Thoracic central venous occlusion (TCVO) can be classified into 4 types.
• TCVO is common in patients with a history of central venous catheters or transvenous pacemaker leads, with a prevalence of up to 50%.
• The inside-out technique is a safe and highly effective technique with a success rate of >90% to re-establish access in SVC occlusion.
• Conduction system pacing is feasible in patients with TCVO using the inside-out technique.

## Introduction

Chronic venous occlusion is an increasingly common problem faced during transvenous cardiac device implantation or upgrade. This is attributable to the growing number of pacemakers implanted and subsequent need for an upgrade to a complex system (e.g. biventricular pacing, conduction system pacing, implantable cardiac defibrillator). Venoplasty, with or without stenting, and lead implantation on the contralateral side with tunnelling across to the current device site are alternatives. However, venoplasty has variable success rates, and lead implantation on the contralateral side is possible only in the absence of bilateral central venous occlusion or superior vena cava (SVC) occlusion. In this case report, we describe the first case of using the highly effective inside-out method to re-establish SVC access and implant a conduction system defibrillator.

## Case report

A 67-year-old man with SVC occlusion and a background of bilateral nephrectomy and right adrenalectomy for renal oncocytoma, heart failure with reduced ejection fraction type 2 diabetes mellitus, recurrent bilateral pulmonary embolism, chronic thromboembolic pulmonary hypertension, and hypothyroidism was referred to our institution for cardiac resynchronization therapy defibrillator (CRT-D) implantation. He initially presented to his local hospital with a syncopal event, having had 5 other similar syncopal episodes in the last year, following his bilateral nephrectomy for renal oncocytoma. His Holter monitoring at the time of syncope demonstrated broad complex tachycardia consistent with ventricular tachycardia. His transthoracic echocardiogram revealed a left ventricular ejection fraction (LVEF) of 35%–40% (improved from 25%–30% on an echocardiogram 1 year previously). A 12-lead electrocardiogram demonstrated left bundle branch block with a QRS duration of 180 ms. Because of his bilateral nephrectomy, he required hemodialysis 3 times per week. He had a maturing arteriovenous fistula on the left arm and a tunneled central line on the right. The tunneled central line was unintentionally removed by the patient in the hospital when he was showering.

Multiple attempts at transvenous pacemaker implantation from the right side at his local hospital were unsuccessful because of occlusion at the SVC level. It was recommended that we avoid left-sided access by the patient’s renal team because of the maturing arteriovenous fistula. He subsequently underwent screening for subcutaneous ICD but failed in all vectors. Additional attempts at our institution to cross the occlusion from a superior and inferior approach were unsuccessful. After discussion at our multidisciplinary team meeting, we decided to re-establish right SVC access with the inside-out technique using the Surfacer central venous access tool (Merit Medical Systems, South Jordan, UT) and to implant an implantable cardiac defibrillator (ICD) with cardiac resynchronization capabilities.

**Figure 1:**
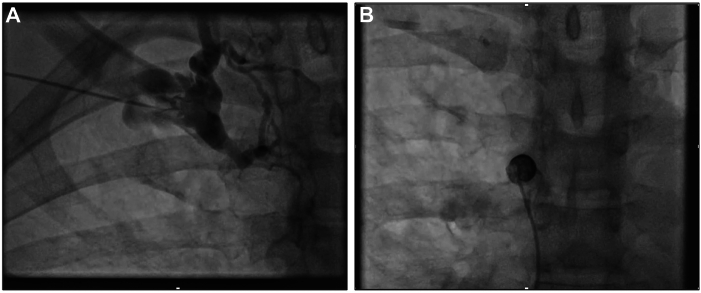
**A:** Contrast injection through an external needle directly into the subclavian vein demonstrating no flow through the superior vena cava (i.e., type 4 thoracic central venous occlusion). **B:** Contrast injection through a long sheath from the proximal end of the superior vena cava demonstrating no flow through.

The inside-out technique involves the use of a workstation sheath, a needle guide and wire (Surfacer device), a radio-opaque circular marker and a peelable sheath. The procedure was performed using general anesthesia because it involved the tunneling of leads, which is uncomfortable, and the patient was primed for emergency surgery in the event of a serious vascular complication. General anesthesia also allowed the use of intraprocedural transesophageal echocardiography to monitor for complications, such as a pericardial effusion. We obtained right femoral vein access under ultrasound guidance. The workstation sheath was then advanced to the right atrium, just proximal to the site of obstruction. A contrast injection was performed, demonstrating no contrast flow through the SVC (Figure 1B), consistent with a type 4 thoracic central vein occlusion as classified by the Society of Intervention Radiology Reporting Standards for Thoracic Vein Obstruction.

**Figure 2:**
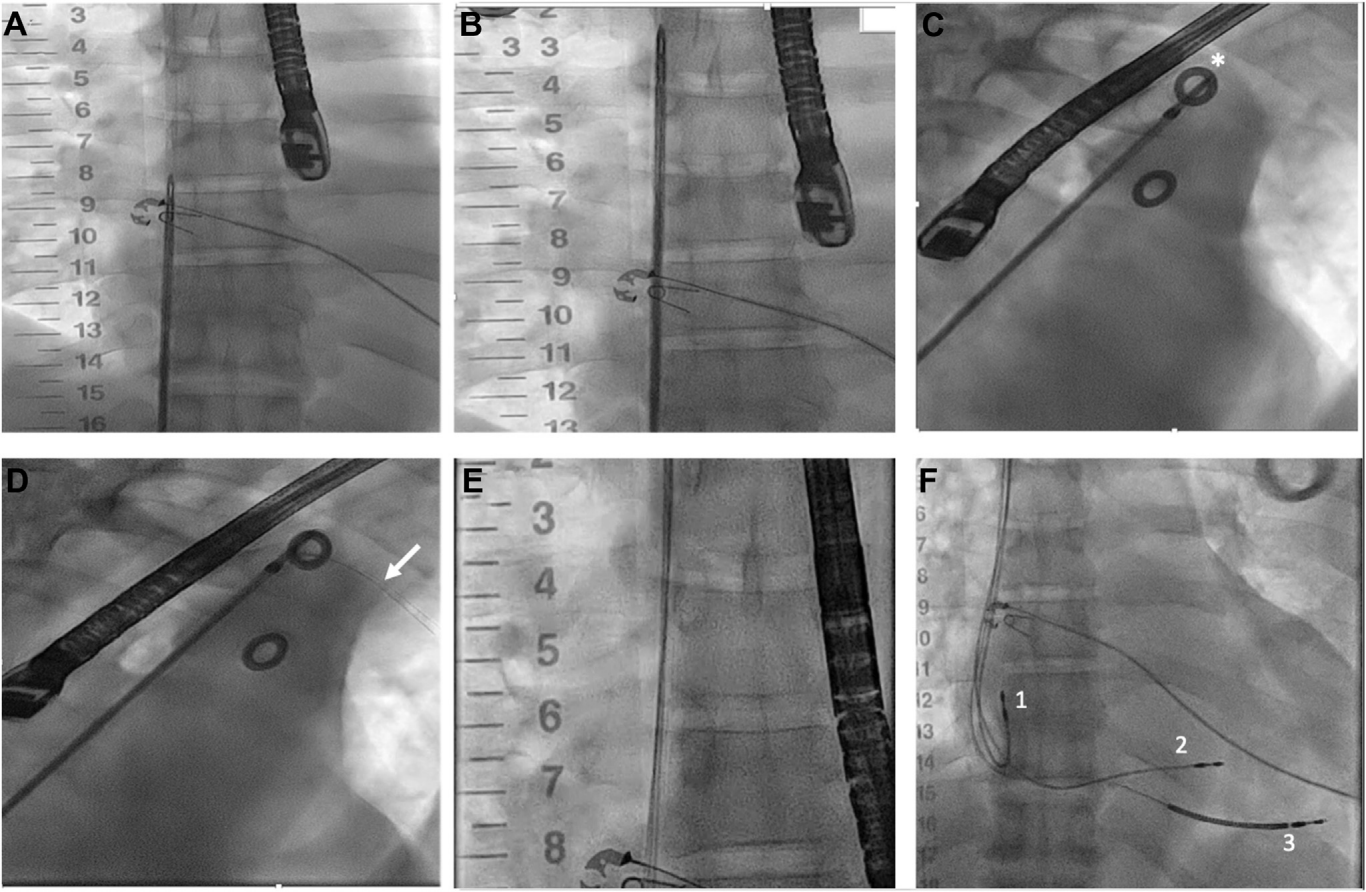
**A:** In posteroanterior (PA) view, the needle guide is advanced to the level of the high right atrium (RA). **B:** In PA view, needle guide is advanced cautiously through superior vena cava obstruction. **C:** Fluoroscopic projection is angulated in the right anterior oblique view until the needle tip appears within the radio-opaque circle (*asterisk*). **D:** The needle wire (*white arrow*) is advanced out anteriorly toward the chest wall and externalized. **E:** Three guidewires are inserted through the peelable sheath into the heart. **F:** Fluoroscopic image at the end of the procedure demonstrating an RA lead (marked *1*), LBBAP lead (marked *2*) and RV ICD lead (marked *3*).

**Figure 3:**
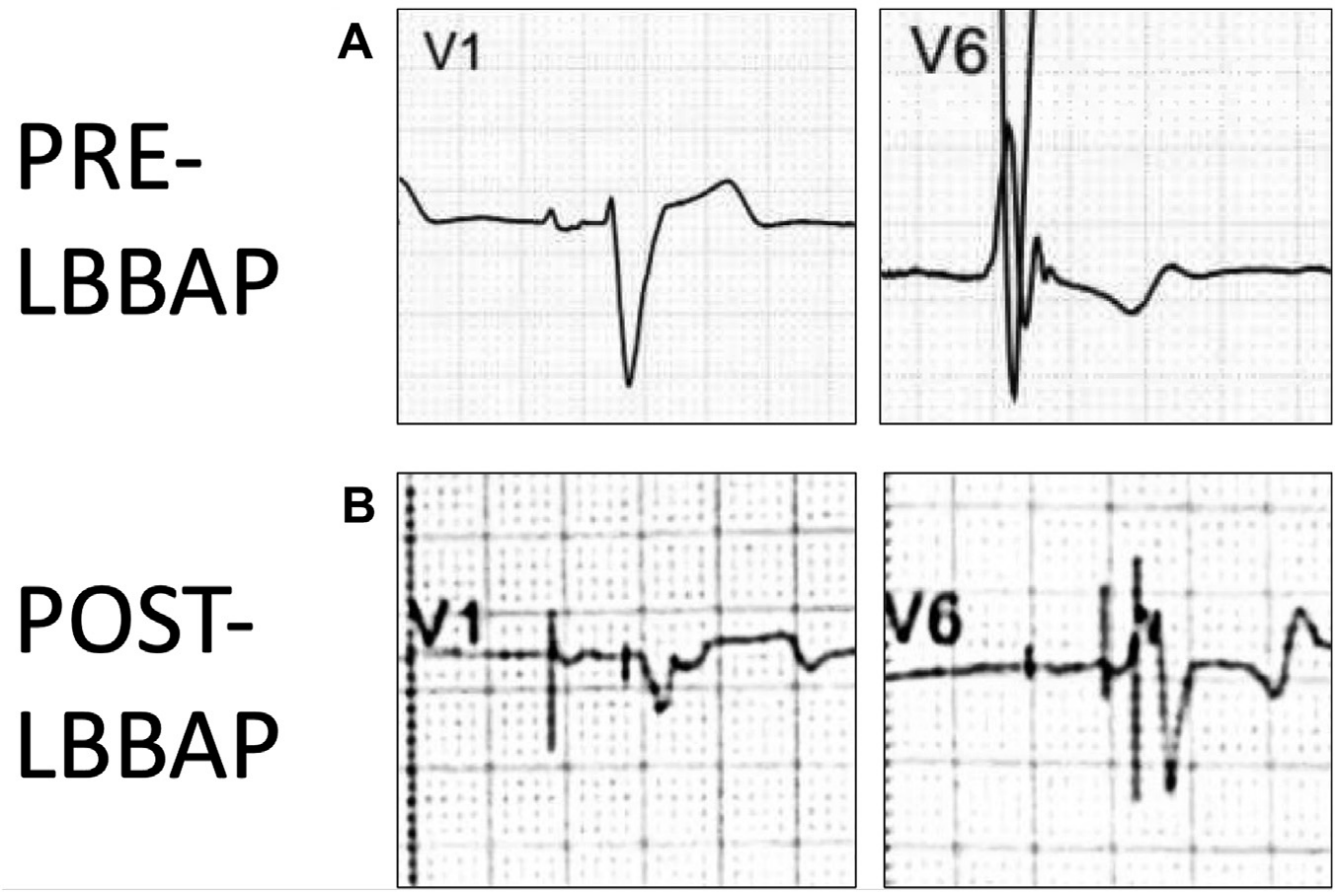
**A:** Leads V1 and V6 before left bundle branch area pacing (LBBAP). **B:** Leads V1 and V6 after LBBAP.

A radio-opaque circle was positioned on the chest wall such that it was within the borders of the contrast-opacified vessel, just above the clavicle, in the posteroanterior fluoroscopic projection. The needle guide and wire were then advanced through the workstation sheath and out of the sheath, cautiously into the occlusion (Figure 2B). The needle guide was advanced further until the tip of the needle was above the clavicle, at the level of the radio-opaque exit circle that was placed on the chest wall. Because of catheter instability at the level below the clavicle and the relatively distal level of SVC obstruction, an infraclavicular exit was not attempted. The fluoroscopic projection was rotated until the tip of the needle appeared within the radio-opaque exit circle (Figure 2C). The fluoroscopic angulation required to achieve this was recorded, and the dial on the proximal end of the device handle was modified to reflect same angulation, ensuring correct alignment of the needle with the exit site. We then carefully advanced the needle wire until it pierced the skin and was externalized enough for the peelable sheath to be exchanged over the wire into the SVC (Figure 2D). At the same time, the needle guide was completely retracted to allow the peelable sheath to enter the occlusion. The needle wire was then removed, and three Terumo guidewires were inserted through the sheath (Figure 2E). We then implanted a ventricular lead (Abbott Tendril STS) into the right ventricular (RV) septum for left bundle branch area pacing (LBBAP) using the Abbott Agilis HisPro delivery catheter, a defibrillator lead (Durata, Abbott, Minneapolis, MN) into the RV apex and an atrial lead (Tendril, Abbott) in the right atrial appendage (Figure 2F). The leads that exited at the supraclavicular level were tunneled to an infraclavicular region where they were connected to a CRT-D device and buried subpectorally. The rest of the procedure was performed as per routine practice. Because of the challenging vascular access in this patient, it was determined that LBBAP was appropriate, and it resulted in good electrical resynchronization. Had this not been achieved, we would have placed a coronary sinus lead. However, with the unusual venous access, it was determined that this would have been technically challenging. Figure 3 illustrates the electrocardiogram before and after LBBAP, demonstrating a qR pattern on V1 and a left ventricular activation time of 75 ms (measured from pacing stimulus to R wave on V6), consistent with successful left bundle branch area capture. The final paced QRS duration was 140 ms, compared with 180 ms before implantation. Supplemental Figure 1 shows chest radiographs in posteroanterior and lateral views after implantation. The patient was discharged a few days later with no complications. Follow-up at 3 months revealed stable lead parameters and significantly improved heart failure symptoms from NYHA III to NYHA II.

## Discussion

To our knowledge, this the first report of the use of the inside-out technique to perform conduction system pacing. We have previously used this technique to achieve conventional cardiac resynchronization therapy (CRT) in another patient with SVC occlusion.^1^ In the previous case, we used an infraclavicular exit versus the supraclavicular exit used in this case. Our patient in this case underwent multiple attempts at superior device implantation which were unsuccessful. Had he passed screening for subcutaneous ICD, he could have undergone subcutaneous ICD implantation instead. However, this would not have provided any resynchronization therapy that would be symptomatically and prognostically beneficial.

Thoracic central vein occlusion (TCVO) is common in patients on hemodialysis and patients with indwelling pacemaker leads. Indeed, between 23% and 46% of patients with indwelling pacemaker leads have a degree of central vein occlusion and 9%–12% have complete occlusion.^2^, ^3^, ^4^ Click or tap here to enter text. Similarly, the prevalence of TCVO in patients with a history of dialysis catheter placement is between 26% and 50%.^5^^,^^6^

The risk of TCVO is increased when subclavian access is used compared to internal jugular access, with an incidence of 42% vs 10%, respectively.^7^Click or tap here to enter text. Furthermore, a longer dwelling time of catheters and high number of catheters used also increase the risk of TCVO.^8^ The pathophysiology of venous occlusion includes endothelial injury, repetitive friction between lead and the vein, disruption to blood flow, inflammation, fibrosis, and activation of the coagulation cascade.

According to the Society of Intervention Radiology Reporting Standards for Thoracic Vein Obstruction, TCVO can be broadly classified into 4 types.^9^ Type 1 is where one internal jugular vein (IJV) or subclavian vein (SCV) is occluded. Type 2 involves occlusion of one brachiocephalic vein (BCV) or ipsilateral occlusion of both the IJV and SCV. Type 3 is where both BCVs are occluded, but there is preserved flow from collateral veins or the azygous vein through the SVC into the right atrium. Type 4 describes SVC occlusion.

Implantation of a transvenous device in patients with TCVO type 3 or 4 is often challenging, as there is no option for implantation of a new lead or pacemaker system on the contralateral side. The options to get around this include subcutaneous ICD, leadless pacemaker implantation via the femoral approach, pacemaker implantation using femoral access, surgical epicardial leads or venoplasty with or without stenting. However, subcutaneous ICDs currently provide only defibrillator therapy with no licensed option for RV pacing or anti-tachycardia pacing via a leadless pacemaker. Pacemaker implantation using femoral access has been associated with up to a 20% rate of atrial lead displacement and surgical epicardial pacemakers have been associated with high rates of lead failure of up to 53%.^10^^,^^11^ Venoplasty with or without stenting, on the other hand, has variable success rates.^12^

The inside-out technique is a safe and highly effective technique to re-establish thoracic vein access, with success rates ranging 90%–100%.^13^ At a level immediately superior or inferior to the clavicle, the subclavian vein is located anteriorly on the chest wall with only fat, muscle, and skin lying anterior to it. An anterior puncture here will avoid puncturing any critical structures such as the lung or arteries, making this technique extremely safe. The first use of the inside-out technique to implant pacemaker leads in the context of TCVO was described by Elayi et al.^14^ At the time, this technique was performed using a transeptal sheath, a modified BRK needle and a sharpened 0.0018-in diameter steel-wire. The initial report included successful re-establishment of access in both left and right TCVOs for transvenous pacemaker lead implantation. To date, based on small observational trials, only one Surfacer device-related acute complication has been reported.^13^^,^^15^ Hentschel et al.^15^ described a case of pericardial perforation secondary to operator error, where the workstation sheath was repositioned after its initial position was confirmed on fluoroscopy, resulting in an erroneous impression of its ongoing position.Click or tap here to enter text.

The inside-out technique has largely been used allow for central venous catheter placements in patients on hemodialysis with TCVO. However, with the rise in pacemaker implantations and the emerging benefits of cardiac resynchronization therapy, it is likely that more device upgrade procedures will need to be performed in the future in the patients with TCVO. We are the first to report the successful use of the inside-out technique for conduction system pacing. In our case, we were able to implant three leads including an atrial lead, RV pace/sense lead and a defibrillator lead. Our study demonstrates that patients with TCVO, including SVC occlusion, should not be precluded from undergoing conduction system pacing as the inside-out technique is safe and highly effective at re-establishing thoracic central vein access. Moreover, manipulation of the lead to achieve left bundle branch area capture is feasible once access is re-established. This technique is therefore a skill that a complex device implanter may benefit from having in their armamentarium.

## Conclusion

In this case report, we demonstrated that the inside-out technique is safe and feasible, and it can be used to re-establish venous access in TCVO for conduction system pacing.

## Disclosure

The authors have no conflicts of interest to disclose.
