# Supplementary material for: “Inside-out” technique to allow conduction system pacing in superior vena cava obstruction
Source: HeartRhythm Case Rep. 2024 Nov 12;11(2):150–4. doi: 10.1016/j.hrcr.2024.11.002 (PMC11862144; doi:10.1016/j.hrcr.2024.11.002)
Supplement: Supplemental Figure 1 [file mmc1.docx]

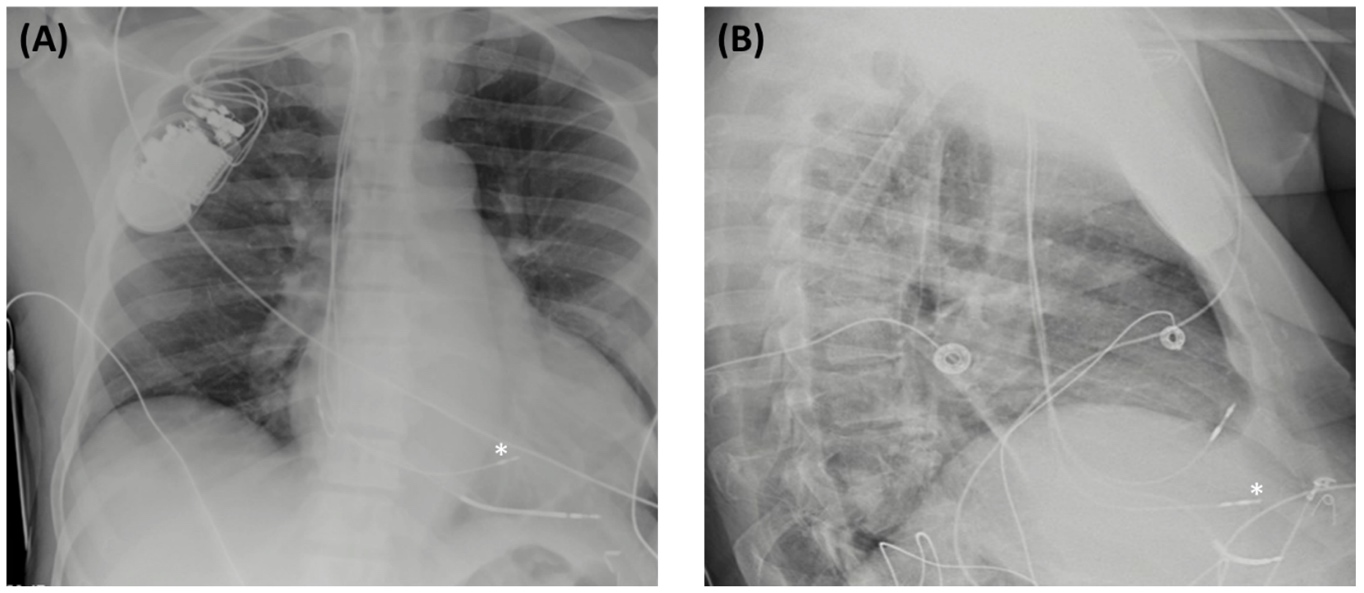


Supplemental Figure 1. (A) PA chest X-ray post-implantation, (B) Lateral chest X-ray post-implantation, confirming septal position of LBBAP lead (marked *).
